# Effect of Tan Tui combined with kinesio taping on the posture control of patients with PFPS: protocol for a randomized controlled trial

**DOI:** 10.1186/s13063-023-07465-z

**Published:** 2023-08-09

**Authors:** Youhua Li, Shuai Tian, Lu Jin, Jixin Li, Xianfa Liu, Jingjing Ji

**Affiliations:** Department of Physical Education, Southeast University, Nanjing Jiangsu, 211189 China

**Keywords:** Patellofemoral pain syndrome, Wushu, Physiotherapy, Randomized controlled trial

## Abstract

**Background:**

Patellofemoral pain syndrome (PFPS) is a chronic disease. Its early symptoms are mild and can be relieved by rest after the pain. If there is no effective rehabilitation, it may develop into patellofemoral arthritis. Physiotherapy and appropriate exercise intervention can improve PFPS and postural control during exercise. Tan Tui (TT) is an effective means to improve postural control. Whether combined kinesio taping (KT) can be used as an effective treatment for PFPS patients’ recovery has not yet been confirmed.

**Methods/design:**

Seventy-two eligible patients with early-stage PFPS will be recruited and randomized into 4 groups: TT + KT group (*n* = 18), TT + KTp group (*n* = 18), KT group (*n* = 18), and CON group (*n* = 18). The TT + KT group was treated with TT combined with KT intervention; the TT + KTp group was treated with TT and KT placebo technical intervention; the KT group was treated with KT intervention alone; the CON group was treated with routine activities. All 4 groups received 30 min, three times a week, for a total of 6 weeks of intervention training. Measurements will be performed at baseline, mid-intervention (4 weeks), and post-intervention (6 weeks) with visual analog scale/score, (VAS), Knee joint Lysholm function score (Lysholm), UniPedal Stance Test (UST), Star Excursion Balance Test ( SEBT), Relative Peak Torque, (RPT), and Knee joint Position PercePtion (KJPP), to check the maintenance of the effect of any intervention.

**Discussion:**

For the first time in this trial, the impact will be evaluated. If the results are the same as expected, they will provide evidence that TT combined with KT sticking intervention can promote the posture control of patients with early PFPS.

**Trial registration:**

Chinese Clinical Trial Registry ChiCTR2100051166. Registered on 15 September 2021.

**Supplementary Information:**

The online version contains supplementary material available at 10.1186/s13063-023-07465-z.

## Background

Patellofemoral pain syndrome (PFPS) refers to a musculoskeletal pain condition caused by patellofemoral joint activity [1]. It is the most common 20.65% of 10–35-year-old sports youth [2, 3]. The main symptoms are anterior knee pain, rubbing noise in the patellofemoral joint, and soft legs, which are further aggravated during physical activity [4], which not only affects the quality of life of patients, but also increases their medical costs [5]. Studies have shown that the early symptoms of PFPS are mild and can be relieved by rest after the pain. If there is no effective rehabilitation, it may develop into patellofemoral arthritis, which will have a long-term impact on the patient’s knee joint health [6]. At present, there is no academic consensus on the occurrence mechanism of PFPS, and most people believe that it is a combination of various factors. It is mainly related to abnormal patella position [7], insufficient gluteal and quadriceps muscle strength [8], abnormal soft tissue tension around the knee joint [9], and patellofemoral joint overload [10].

At present, PFPS interventions are numerous and gradually standardized. It focuses primarily on exercise therapy and has been shown to relieve pain and improve function in the early, middle, and long term [11]. Exercise therapy includes quadriceps exercises [12], hip strength exercises [13], stretching exercises for tissues around the knee joint [14], gait retraining [15], and core muscle exercises [16]. A systematic review of studies discussing the effectiveness of interventions in patients with PFPS reported consistent evidence of pain reduction and functional improvement after several weeks of exercise therapy [17–20].

In addition to exercise therapy, other interventions for PFPS have also achieved significant results, such as kinesio taping (KT). Patellar taping was first proposed by Moconel, which uses taping to pull the patella from the lateral side of the knee to the medial side of the knee to correct the abnormal trajectory of the patella [21]. On this basis, Kinesio proposed the KT technique, which can reduce pain in patients with PFPS [22], improve lower extremity function [23], and increase the knee flexion angle to improve lower extremity postural control [24]. Studies have shown that KT can improve functional abnormalities and enhance quadriceps muscle strength in athletes with PFPS [25], but the results of a systematic review by Barton et al. showed that the use of KT technology could reduce knee pain in the short term, but not in the long term [26]. Therefore, more studies are needed to verify the positive effect of KT on PFPS patients.

Wushu is known as “Southern Boxing and North Legs.” Tan Tui (TT) is a typical example of the Northern School’s proficient leg technique. It is characterized by fast and powerful, left and right symmetry, simple shape, easy to learn, and neat power frame [27]. It is a classic essential skill exercise of Chinese martial arts, known as the “Beginner’s Guide to Playing TT.” After practice, it will have a positive impact on the practitioner’s strength and stability, and other physical qualities. At the same time, it can stabilize the footwork of the lower limbs, and all parts of the body can be developed in a balanced way. When it is static, it is straight and stretched, and when it is moved, it is quick to shoot, which plays a vital role in the control of body posture [28, 29]. At the same time, the ancient Chinese classics of boxing pointed out that the effect of TT is “increasing vigor, steady posture, practicality, and strengthening muscles and bones,” which can significantly improve the lower limb strength and balance ability of practitioners [30]. Professor Zhang Wenguang’s book on sparring also holds the same point of view [31]. Therefore, TT is an effective means to improve the ability of postural control, but intervention for PFPS patients is rare at present, especially the intervention for Taijiquan PFPS patients.

Currently, there is insufficient evidence to demonstrate the effect of TT or combined with KT on the efficacy and postural control of patients with PFPS. At the same time, the longer the duration of patellofemoral pain, the less successful the treatment will be [32]. Therefore, this paper takes PFPS patients as the intervention object and observes the effect on the curative effect and posture control of PFPS patients through TT or combined KT intervention. Evidence support for prevention.

## Methods/design

### Study purposes

The purpose of this article was to observe the effect of 6-week TT or combined KT on the efficacy and postural control of patients with early PFPS.

### Study design

This is a single-blind randomized controlled trial that recruited 72 eligible early PFPS patients and randomly divided them into 4 groups: TT + KT group, TT + KTp group, KT group, and CON group with a ratio of 1:1. The TT + KT group was intervened with TT combined with intramuscular tape; the TT + KTp group was intervened with TT and intramuscular tape with placebo technical intervention; the KT group was intervened with intramuscular tape alone; the CON group was performed with routine activities. Measurements will be taken at baseline, mid-intervention (4 weeks), and post-intervention (6 weeks) to examine the maintenance of any intervention effects.

### Patient public involvement

**Fig. 1:**
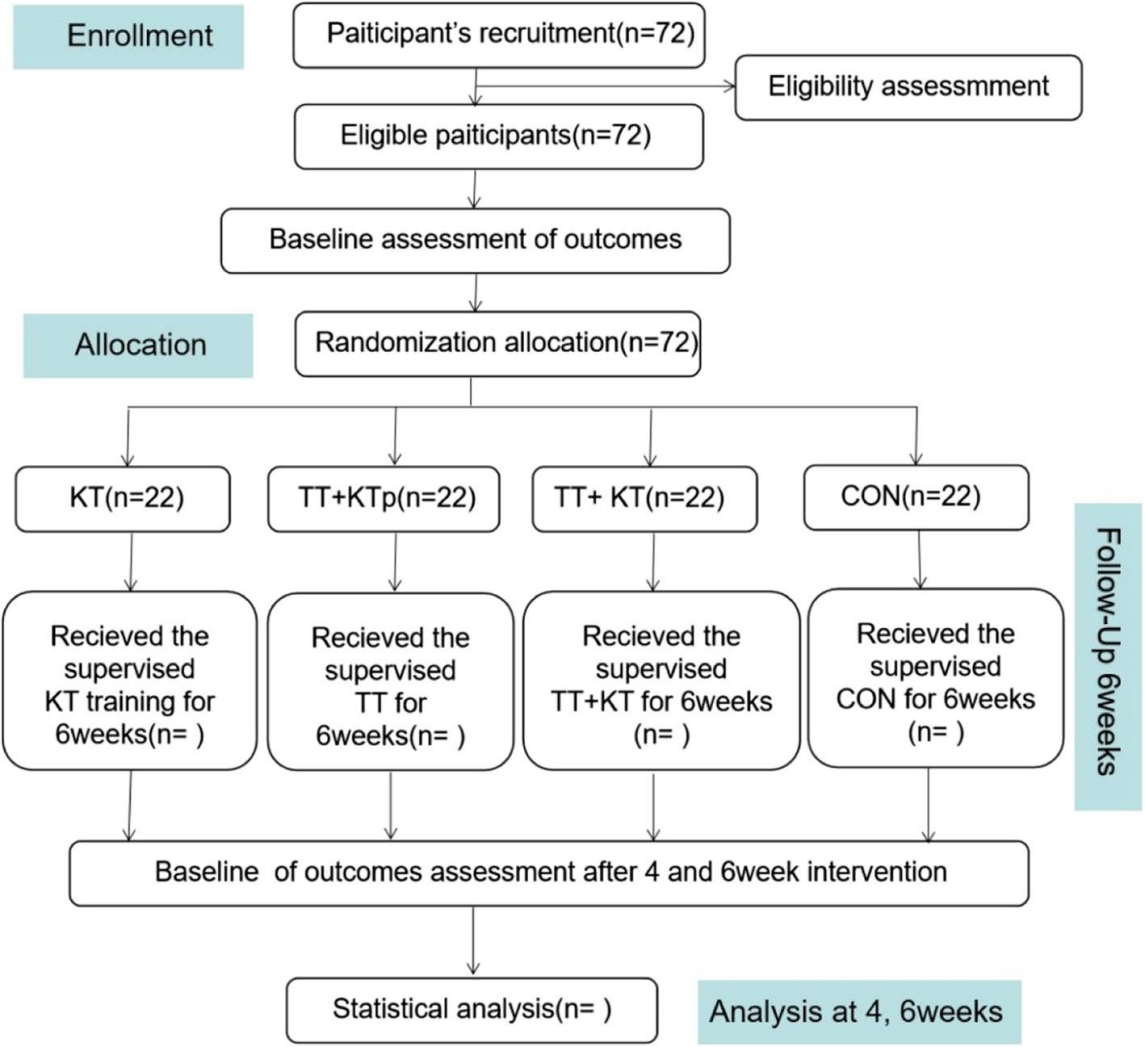
Proposed participant flow. TT + KT, kinesio taping + Tan Tui; TT + KTp, placebo kinesio taping + Tan Tui; KT, kinesio taping; CON, control group

The participant flow for this trial is shown in Fig. 1. This protocol follows the Standard Protocol Items: Recommendations for Interventional Trials (SPIRIT) guidelines and meets the SPIRIT checklist (see Additional file 1).

### Sample size calculation

The sample size was estimated by the software G*Power 3.1.9 [33]. According to the experimental design of 4 (group) * 3 (time) in this study, repeated measures analysis of variance was used to test the research hypothesis. Under the effect size of 0.25, α of 0.05, and power of 0.8, the sample size requirement was 64 people. After considering the dropout rate of 10%, the overall sample size was calculated to be 71 people. At the same time, combined with the proportionality of the sample size between groups, the final sample size was determined to be 72 people, and each group was about 18 people.

### Setting and recruitment

This study will recruit PFPS patients through WeChat, flyers, and Weibo in the Football Academy of Beijing Sports University, China. The exercise intervention was independently supervised by 3 research assistants at the University, and the other research assistants independently conducted data collection. Due to the impact of the new crown epidemic, this study will officially start recruitment in October 2022.

### Participant eligibility

PFPS patients were recruited for sports-specialized college students at a university in Beijing. Diagnostic criteria [34–36] are as follows: (1) retropatellar or peripatellar pain for ≥ 4 weeks; (2) at least two induced pains when completing functional activities such as hopping on one foot, going up and down stairs, squatting, running, kneeling, and bending knees for a long time; (3) VAS score ≥ 2 when the pain was the most severe in the last week; (4) Lysholm score < 70; (5) at least two of the following signs were positive: tenderness on the patella or patella circumference, crepitus on the patellofemoral joint surface, Fear test, patellofemoral grinding test, knee extension resistance test, single foot squat test. Inclusion criteria were (1) aged 18–30 years; (2) met the diagnostic criteria and had unilateral involvement; (3) did not receive medication or physical therapy; (4) voluntarily signed informed consent. The exclusion criteria were: (1) damage to the tissues around the knee; (2) structural damage to the knee, such as fracture, meniscus tear, or osteoarthritis; (3) allergy to KT; (4) physical activity Medical contraindications (such as cardiovascular disease); and (5) Inability to adhere to the intervention due to personal or other reasons. All subjects voluntarily signed a written informed consent form before the intervention and were given an explanation of the research process by the researchers, strictly following the ethical standards of the Declaration of Helsinki.

### Randomization, allocation, and blinding

In this trial, the assessments and treatments were performed by different therapists. The evaluator was blinded to the subjects’ assignment. All the intervention procedures were performed by the same physiotherapist with experience in the field of sports physical therapy. Both the physiotherapist and the participants were blinded to the purpose of the study. In addition, a different researcher, blinded to the object of the study, carried out the data analysis.

## Intervention

### Intervention plan

#### Kinesio taping

KT function correction technology is a kind of KT technology [37]. The width of the patch is 5 cm, the thickness is 0.05 cm, and it has 70% tension. It is pasted on the skin of the quadriceps muscle, and the natural tension is used to move along the muscle to the tendon, so as to maximize the flexion of the knee joint, the two caudal ends of the muscle tape meet down the tibial tuberosity along both sides of the patella. The calculation of the percentage of tension is based on the length of the KT tile on the paper (0%), and the tape is stretched at maximum tension to measure its length (100%). Therefore, the tension required for KT in this study was 70%, which is 70% of the length between the maximum available tension (100%) of the tape and the base point (0%) [38]. After the taping was completed, the subjects were asked whether there was pain, and they performed half-squatting movements to compare the pain, and the taping was completed if there was no pain.

#### Placebo kinesio taping

The application of the patch was the same as before, but the tension of the patch was 0%, and the patch was only attached to the ankle joint of the subject without any stretching. Both functional correction taping and placebo taping techniques were applied to the subject’s bilateral quadriceps and taped once every 5 days [39, 40].

#### Tan Tui

**Fig. 2:**
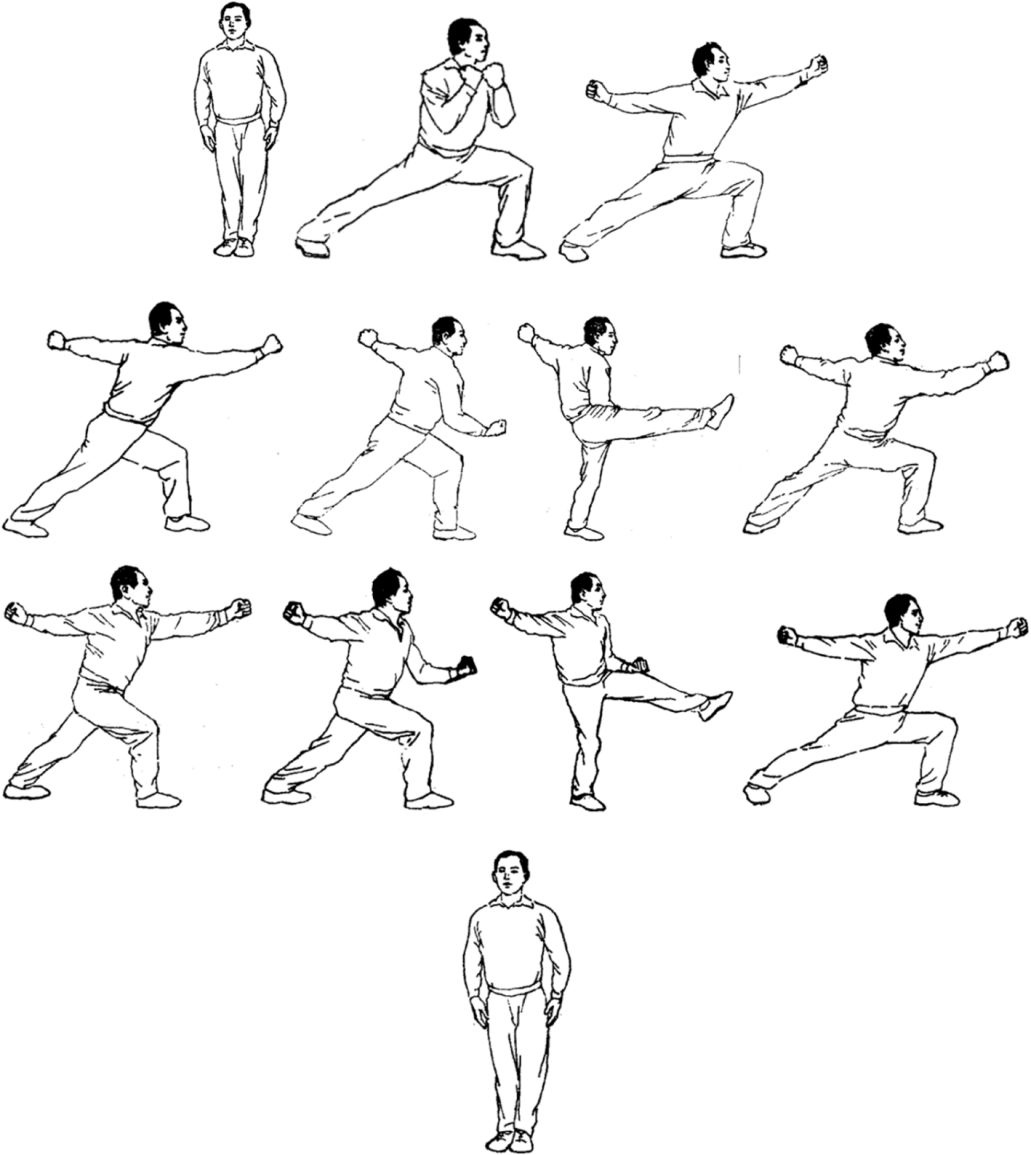
Tan Tui training movement [31]

The selection of the TT exercises is based on the physiological characteristics and physical fitness status of the students in the Taijiquan special class. Through the classification and statistics of the ten-way TT movements 31 compiled by Professor Zhang Wenguang. The proportions of lunges and leg movements were 61% and 17%, respectively, accounting for 78% of the total number of 10-way TT movements. Therefore, this study selects lunges and slings. The sling exercises include three parts: (1) preliminary movements: standing side by side, lunges clasping fists, and lunges punching; (2) TT exercise movements: right Hengquan, right punch, right leg, right lunge punch, left lateral punch, left punch, left leg, left lunge punch; (3) Closing movements: standing side by side (Fig. 2).

In this study, the intervention cycle of bouncing leg exercise lasted for 6 weeks, 3 times a week, 30 min each time. The intervention was divided into two stages of motor learning in the first 1 week and motor improvement in the last 5 weeks. Movement learning stage: The subjects learn new movements in the first week, and can complete a set of movements independently and basically meet the specifications of each movement. The movement improvement stage in the last 5 weeks: the static time of each movement practice is gradually increased from 1 to 5 s, and the interval time is 1 min, 1.5 min, 2 min, 2.5 min, and 3 min, respectively. Each repetition of the exercise can improve its posture control ability.

The requirements of the leg-bounce exercise: the coordination of the strength of the movement and the breathing should be paid attention to during the practice of each movement of the leg-bounce. The method of each movement follows the law of lower limb exertion that starts from the root, follows the middle, and reaches the slightest. The breathing follows the principle of “lifting the breath” and “supporting the breath” [41], and in terms of technical movement standards, the lunge follows the competition rules of martial arts routines ( 2012) [42], the technical movement of the TT requires the support leg to be upright, the spring kick leg to be level with the ground, and the support leg and the pop-up leg to maintain about 90°.

#### CON

Do regular physical activity as usual.

## Outcome assessment test indicators

### Primary outcome

1. Visual analog scale/score (VAS): VAS was used to evaluate the pain of PFPS patients before and after intervention [43]. On a 10 cm long walking scale, one side has 10 scales, and the two ends are 0 and 10, indicating no pain and the most severe pain, respectively; the other is colored from light to dark, showing different degrees of pain. When using it, turn the scaled side away from the patient, and let the patient mark the corresponding color on the ruler that can represent their pain level, and score [44] according to the position marked by the patient.
2. Knee joint Lysholm function score (Lysholm): Knee joint function is evaluated by the Knee Joint Lysholm Function Score (LKSS), which has been proven to have good reliability and validity [45]. The parameters of the scale (total score 100) mainly include lameness (5 points), twisting (15 points), pain (25 points), support (5 points), instability (25 points), swelling (10 points), Going up and down stairs (10 points), squatting and standing up (5 points), basically covering all symptoms of patients with patellofemoral joint disease. The size of the score is positively correlated with the functional status of the knee joint of patients, and the higher the score, the better the joint function [46].
3. Static balance: The static balance ability of the dominant side of the subjects was assessed by the UniPedal Stance Test (UST) with eyes closed. UST has been shown to have high scoring reliability (ICC = 0.998) [47], and its test refers to the method of previous literature [48]. Before the formal test, the subject’s heart rate should reach a quiet level, and perform 4 test exercises. At the beginning of the formal test, stand on one foot with eyes closed according to the test requirements, and stop timing when the supporting leg is displaced, eyes are opened, or the non-supporting leg touches the ground. Each subject repeated the test 3 times with 20 s rest intervals, and the average of the 3 test scores was the final test score of the subjects.
4. Dynamic balance: The Star Excursion Balance Test (SEBT) was used to evaluate the dynamic balance ability of the dominant side of the subjects. SEBT is a comprehensive quality, including strength, flexibility, and coordination quality, which is widely used to evaluate the dynamic stability of lower limbs [49], and its test method refers to previous studies [50]. Before the formal test, to reduce the test error caused by the learning effect, the subjects performed the test practice 4 times. In the formal test, the subject (left or right foot) is required to stand on one foot, with both hands on the hip, and the non-supporting leg is extended as far as possible in front of the supporting leg, the back-inward direction, and the back-outward direction, respectively, and the non-supporting leg is retracted and close to the supporting leg, then start the next reach. The subject’s supporting leg moved, the non-supporting leg touched the ground, or the non-supporting leg reached quickly and far rather than sustained force during the test was considered a failure. The subject’s measurement in each direction is 3 times, with a 20-s rest between each time. The average of the 3 test data is the subject’s final score in that direction, and the total comprehensive value is the average of the 3 directions. SEBT has been shown to have good internal reliability (ICC = 0.86–0.94)52 [51] and test–retest reliability (ICC = 0.89–0.93) [52].

### Secondary outcome

#### Knee joint isokinetic muscle strength test

The Isomed 2000 isokinetic muscle strength test system in Germany was used to test the isokinetic muscle strength of the dominant side of the subject. After the subjects are fully warmed up, the tester conducts a guided test and conducts adaptive exercises on the test system. In the formal test, a sitting position was used, the thigh and trunk were fixed, the knee joint range of motion was set to be 0–100°, and the test angular velocity was 60°/s. Studies have shown that this angular velocity can better reflect the level of knee flexor and extensor muscle strength [53]. Knee flexion and extension tests were performed 5 times each. The flexor muscles were measured first, and then the extensor muscles were measured. The dominant leg was tested first and then the non-dominant leg was tested. The interval between each test was 60 s, and the average was taken as the final test score. The test index is Relative Peak Torque (RPT), representing the peak torque per unit body weight. The larger the RPT value, the greater the knee joint muscle strength.

#### Knee joint proprioception test

The German Isomed 2000 isokinetic muscle strength test system was used to test the subject’s dominant knee joint position perception (Knee joint Position PercePtion, KJPP) evaluation. The knee joint position awareness test adopts the passive position awareness reduction method, and the target angle is 45° [54] of knee joint flexion. During the formal test, the subject’s knee joint was driven by the instrument to the target angle, and then the subject felt the angle for 5 s and returned to the starting position. Then, the knee joint was driven by the instrument at a speed of 1°/s. When the subject felt that the target angle was reached, the brake switch was quickly pressed to record the actual angle of the subject. The subject’s position sense is the absolute value of the difference between the actual angle of its reset and the target angle. The smaller the value, the better the position sense.

### Safety measurements

Any unexpected adverse events that occurred during the 6-week intervention period will be reported to the research assistant, and the causal relationship between y and Tan Tui exercises and kinesio taping effects will be evaluated. If a severe sports injury or other adverse event occurs, the research assistant will immediately report to the project leader and the Sports Science Ethics Committee of Beijing Sport University; they will decide whether the participant needs to withdraw from the study.

### Data collection

Subject demographics will be collected during the recruitment process. Data on primary and secondary outcomes will be collected by professional outcome assessors at baseline, at 4 weeks of intervention, and at 6 weeks of intervention. All outcome assessors had standardized training on test methods prior to intervention to ensure that all subjects had the same test conditions. To ensure the attendance of students and provide more complete data results, we will provide all students with free professional TT teaching services, free knee pain treatment, and a reward of 100 yuan. It will be released via WeChat after 6 weeks of intervention.

### Data management

The primary and secondary results of the test will be recorded through the case report form (p-CRF), and the paper version of the data will be processed electronically through the free data management software EpiData Manager on time. Two result evaluators separately reviewed and confirmed the data, and converted it into a format that can be used for statistical analysis.

### Statistical analysis

Statistical analysis will be performed by the Statistical Package for Social Sciences (The Statistical Package for Social Sciences, SPSS 23, SPSS Inc., Chicago, IL, USA). ShaPiro Wilk will be used to test whether the variables of the two-way multivariate analysis of variance conform to the normal distribution. One-way multivariate analysis of variance will be used to compare the baseline age, height, and weight data of participants, to explore whether there is homogeneity between groups. Descriptive statistical analysis will be conducted in the form of mean ± standard deviation. Two-way multivariate analysis of variance will be used to perform inference analysis on the effect of the intervention group (e.g., TT + KT, KTp + TT, KT, CON) and time (e.g., pre-intervention, during intervention, post-intervention), in order to examine group effect and time effect and The interaction effect of time and group. Pairwise comparisons of variables with significant effects in ANOVA will be conducted by post hoc analysis and Bonferroni correction to find time effects by group and group effects by time. According to Cohen’s method, the effect size will be divided into large (0.8), medium (0.50–0.79), and small (0.20–0.49) to judge the significance of the intervention effect [55]. *P* < 0.05 means there is a significant difference, and *P* < 0.01 means the difference is extremely significant.

## Ethics

The conduct of this research will comply with the principles of the Declaration of Helsinki, and relevant ethical guidelines, including informed consent and confidentiality, and data storage. The ethics were approved by the Ethics Committee of Beijing Sport University Sports Science trial (Approval Number 2021125H). All participants will be fully informed of the trial situation and sign an informed consent form before participating.

## Monitoring

Tan Tui is an aerobic exercise with low risk. This study is not expected to cause any potential harm. Therefore, there will be no data monitoring committee, interim analysis, or stopping rules. We do not anticipate any potential harm. Therefore, there will be no Data Monitoring Committee, interim analyses, or containing rules.

## Dissemination

The research protocol has been registered and viewed on the China Trial Registration website (registered at ChiCTR.org, with the identifier ChiCTR2100051166). The research results will be disseminated to all participants, researchers, healthcare providers, and sponsors through research summary documents, courses, presentations, and the Internet. The research will also be published in scientific journals and presented at conferences, targeting a wide range of groups.

The results will be disseminated to all participants, researchers, healthcare providers, and sponsors through study summary documents, courses, presentations, and the Internet. This study will also be published in scientific journals and presented at conferences to target a wide range of groups.

## Discussion

The primary purpose of this study was to observe the effect of TT or combined with KT on the curative effect and postural control of patients with PFPS. The related parameters of curative effect were pain and knee function; the associated parameters of postural control were static balance, dynamic balance, knee muscle Force, and knee proprioception. The best method to improve the curative effect and postural control ability of PFPS patients has not yet been obtained [56]. The intervention in this paper will provide a new method for the rehabilitation of PFPS patients.

Studies generally believe that intervention in the knee joint of patients with PFPS can significantly improve patellofemoral pain and knee function. Some studies have performed knee strength training alone or combined with hip strength training in patients with PFPS and found that both interventions can achieve the effect of reducing pain and improving function, but the effect of the hip joint combined with knee strength training group is better than that of the simple group. Knee Strength Training Group [13, 57]. Valenza et al. randomly divided 84 patients with PFPS into a soft tissue stretching group, a transcutaneous electrical stimulation group, and a control group, and compared the immediate effects of the two interventions on the range of motion and pain of the knee joint. The results showed that both groups improved the range of motion of the joint, and reduced pain, but the soft-tissue stretch group had better treatment effects [14]. The characteristics of the TT exercise are mainly the flexion and extension of the hip and knee joints. When the joint is extended, it is supported by one leg. When the other leg pops out and performs a short-term isotonic contraction, it can effectively stimulate the muscles around the hip and knee joints. At the same time, it can quickly stretch the soft tissues around the knee joint at the moment of popping up; when the joint is flexed, it is performed as a lunge action, and the quadriceps of the front leg performs isotonic contraction to enhance its muscle strength. It can be seen that the TT can not only improve the strength of the hip and knee joint, but also enhance the flexibility of the soft tissue around the knee joint.

PFPS patients mainly occur in sports groups. Their performance is affected by the inability to maintain the stability of specific postures in technical movements due to knee pain and functional loss. Therefore, postural control is particularly important for patients with PFPS in the athletic population. Balance ability is the external manifestation of postural control, while muscle strength and proprioception are the internal mechanisms of postural control. Studies generally agree that postural control in patients with PFPS can be significantly improved through exercise intervention or combined with physical therapy. The study by Zarei et al. conducted 6-week exercise therapy and exercise therapy combined with dry needling intervention in 40 female athletes and observed their pain, function scores, and dynamic balance tests at baseline, 4 weeks, and 6 weeks of intervention. The results showed that exercise Therapy and exercise therapy combined with dry needling interventions significantly improved female athletes’ homeostasis after 4 and 6 weeks [35]. In a study by Baldon et al., 8 weeks of quadriceps exercises and a stabilization exercise consisting of hip, lower extremity, and trunk in patients with patellar pain syndrome were found to be more effective than quadriceps exercises alone, kinematics, and hip-knee strength were better [58]. Different exercise rehabilitation therapies have shown positive effects on postural control or its components in patients with PFPS, and a panel of experts at the Fifth International Patellofemoral Pain Research and Rehabilitation Centre in Australia recommended the use of exercise therapy to improve function in patients with PFPS [59], especially for the knee. Stretching exercises for strengthening and flexibility of the muscles around the joints and hip joints, as well as stabilization exercises for the entire body, have been shown to be effective [60, 61].

The TT exercises in this article include multi-dimensional physical qualities such as strength, flexibility, and speed. The TT exercise is essentially a muscle isometric contraction of the knee joint in flexion or extension and is consistent with the functional movement of the knee joint. The core impact of Chinese martial arts on human health is to improve its stability. As an ancient boxing type of traditional Chinese martial arts, bouncing legs is an introductory routine for practitioners. Its core function is to improve the stability of practitioners. As stated in the ancient Chinese classics of boxing, “growing strength and steady posture” can significantly improve the strength and stability of the lower limbs of practitioners [30].

At present, the effect of KT on postural control ability has not been uniformly concluded. Some studies have shown positive results. Some scholars have reported that the use of myotropic patches in the pain area can increase the effect of fast afferent fibers, thereby inhibiting the transmission of pain perception to the brain, thereby increasing the pain threshold and reducing pain [62]. The Mason study showed that the application of KT could significantly improve the angle of painless flexion of the knee joint and improve the postural control of the lower limbs [24]. Logan et al. conducted a systematic review of the effect of KT on PFPS patients, including 5 randomized controlled trials with a total of 235 PFPS patients. The results showed that KT alone has a small effect and can only be used as an adjunct to exercise therapy [63]. Therefore, the effect of KT on the postural control ability of patients with PFPS still needs further research to confirm.

## Trial status

Due to the impact of the novel coronavirus, the experiments in this study have not yet started. The research experiment is expected to begin recruiting subjects in October 2022 and end in mid-November 2022. Testing and intervention training will be conducted from the end of November 2022 to mid-January 2022.

### Supplementary Information

**Additional file 1.** SPIRIT checklist.

## Data Availability

Data for the study can be made available upon request. Interested researchers should contact Dr. Li at li2513436@126.com.

## Abbreviations

KT: Kinesio taping
TT: Tan Tui
KTp: Placebo kinesio taping
UST: Unipedal Stance Test
SEBT: Star Excursion Balance Test
RPT: Relative Peak Torque
KJPP: Knee joint Position PercePtion
