# Correction: Efect of Tan Tui combined with kinesio taping on the posture control of patients with PFPS: protocol for a randomized controlled trial

**DOI:** 10.1186/s13063-023-07675-5

**Published:** 2023-10-03

**Authors:** Youhua Li, Shuai Tian, Lu Jin, Jixin Li, Xianfa Liu, Jingjing Ji

**Affiliations:** 1 Department of Physical Education, Southeast University, Nanjing, 211189 Jiangsu China; 2 Liaoning Police College, Ganjingzi District, Dalian, Liaoning China; 3 Central University of Finance and Economics, Changping District, Beijing, China; 4 Suzhou Vocational University, Wuzhong District, Suzhou, Jiangsu China; 5 Henan University of Technology, Zhengzhou, Henan China

**Correction: Trials 24, 507 (2023)**

**https://doi.org/10.1186/s13063-023-07465-z**

Following publication of the original article [1], we have been notified that the author affiliations are incorrect.

Correct affiliations are below:

Youhua Li, Southeast University

Shuai Tian, Liaoning Police College

Lu Jin, Central University of Finance and Economics

Jixin Li, Suzhou Vocational University

Xianfa Liu, Henan University of Technology

Jingjing(CA) Ji, Southeast University
